# Genome-wide identification and analysis of monolignol biosynthesis genes in *Salix matsudana* Koidz and their relationship to accelerated growth

**DOI:** 10.48130/FR-2021-0008

**Published:** 2021-04-23

**Authors:** Guoyuan Liu, Yixin Li, Yu Liu, Hongyi Guo, Jiaming Guo, Yawen Du, Yanhong Chen, Chunmei Yu, Fei Zhong, Bolin Lian, Jian Zhang

**Affiliations:** Key Lab of Landscape Plant Genetics and Breeding, School of Life Science, Nantong University, Nantong, China

**Keywords:** lignin biosynthesis, *Salix matsudana* Koidz, accelerated growth, evolution, RNA sequencing

## Abstract

Lignin plays an important role in plant growth and development. It serves as a raw material for the manufacture of paper, animal feed, and chemical fertilizers. However, the regulation of lignin biosynthesis genes and the composition of the relevant gene families remain unclear in many plant species. Here, we identified and characterized 11 families of monolignol biosynthesis genes in *Salix matsudana* Koidz. Based on phylogenetic analysis of lignin biosynthesis genes from nine angiosperm species (*Arabidopsis thaliana*, *Oryza sativa*, *Zea mays*, *Solanum lycopersicum*, *S. suchowensis*, *S. purpurea*, *Populus euphratica*, *P. trichocarpa*, and *S. matsudana*), the 11 gene families could be divided into two classes that differed in their apparent evolutionary history. We compared the distribution of lignin biosynthesis genes between the two sub-genomes (At and Bt) of *S. matsudana* and found that more duplicated genes were present in the Bt sub-genome. We analyzed RNA sequencing data from two parents of contrasting height and two of their F_1_ progeny, and detected 23 differentially expressed genes (DEGs) that may regulate accelerated growth. We analyzed the promoter regions of the lignin-related DEGs and identified several hormone-related (auxin, ethylene, and cytokinin) transcription factor binding sites. These results provide an important foundation for future studies on the molecular mechanisms and genetic regulation of lignin biosynthesis and its relationship to accelerated growth in forest trees.

## INTRODUCTION

Lignin is an aromatic phenolic compound found in the cell walls of vascular plants and accounts for 18–35% of total plant biomass^[1]^. Lignin is deposited in the walls and intermediate layers of some vascular plant cells, where it fills in the spaces of the cellulose microfibril framework. Lignin enhances the mechanical strength of plant cell walls, and its hydrophobicity enables the long-distance transport of water and nutrients in the xylem^[2]^. Lignin also serves as a defensive barrier against insect pests and can protect plants from bacterial infections^[3]^. It is used in the production of engineering plastics, thermoplastic elastomers, and polymer foams^[4]^. Lignin content is a critical factor that determines the application of specific woods. Wood with low lignin content is easier to degrade and is often used in the pulp and paper industries, whereas wood with high lignin content has stronger mechanical properties and is often used for construction and decoration^[5]^.

Lignin is polymerized from the three monolignols *p-*coumaryl alcohol, coniferyl alcohol and sinapyl alcohol, also called the H, G and S subunits. These aromatic alcohols are produced from phenylalanine and tyrosine via the phenylpropanoid pathway through a series of reactions that include deamination, hydroxylation, methylation, and reduction^[1]^. Enzymes involved in this process include 4-coumarate:CoA ligase (4CL), cinnamate-4-hydroxylase (C4H), phenylalanine ammonia lyase (PAL), caffeic acid 3-*O*-methyltransferase (COMT), hydroxycinnamoyl-CoA shikimate/quinate hydroxycinnamoyl transferase (HCT), cinnamate-3-hydroxylase (C3H), caffeoyl shikimate esterase (CSE), caffeoyl-CoA 3-*O*-methyltransferase (CCoAOMT), cinnamoyl-CoA reductase (CCR), cinnamyl alcohol dehydrogenase (CAD), and ferulate-5-hydroxylase (F5H). The resulting monolignols are polymerized by oxidases (peroxidases, POX) and laccases (LACs) to form lignins that contain different amounts of the three monolignols.

Lignin content and composition differ among plant species. In general, herbaceous plant lignin contains G, S, and H subunits, hardwood lignin contains mainly G and S subunits, and coniferous wood lignin consists almost entirely of G subunits^[6]^. In angiosperms, lignin is composed mainly of G and S monolignols, along with much lower amounts of H subunits. Bonawitz and Chappie (2010) contend that S-type lignin provides greater flexibility and that this flexible polymer may be important for herbs that regrow their aboveground biomass each year^[7]^.

Key genes that encode essential enzymes for monolignol biosynthesis have been identified in a number of plant species^[1,4,8]^. As more plant genomes become available, genome-wide surveys enable a systematic characterization of key enzymes and their corresponding family members. Lignin plays a crucial role in the evolution of plant species from aquatic algae to terrestrial plants^[9]^. Additional research is needed to better understand the origin and biosynthesis of this important plant polymer^[10]^. Identification and functional characterization of lignin biosynthesis enzymes and their associated genes will provide a foundation for the systematic analysis of carbon flux through lignin metabolism. Such research can guide the genetic improvement of forestry species^[11]^.

Due to its strong tolerance to salt, heavy metal, and cold damage and its resistance to diseases and pests, *Salix matsudana* Koidz has a wide global distribution, especially in China. Willow produces large amounts of biomass, and it is easy to propagate and is rich in variety. It is widely used in commercial forestry, and its wood is an important raw material for paper, gunpowder, construction equipment, particleboard, and other industrial products. The release of the *S. matsudana* genome now enables the identification of key lignin biosynthesis genes and gene families. Although there have been a number of reports on monolignol biosynthesis genes in *Populus*^[12]^, most studies have focused on one or two genes rather than considering whole gene families.

The systematic identification and functional annotation of key monolignol biosynthesis genes in *S. matsudana* provide important background data for improving its lignin content and composition^[13]^ to obtain fast-growing and high-quality wood. The results also provide insight into the evolution of the monolignol biosynthesis pathway at the molecular level.

## MATERIALS AND METHODS

### Identification of monolignol biosynthesis genes

The monolignol biosynthesis pathway (map00940) was retrieved from the KEGG pathway database (https://www.kegg.jp/kegg/pathway.html). Related genes from the reference genomes of *A. thaliana*, *O. sativa*, *Z. mays*, *S. lycopersicum*, *P. euphratica*, and *P. trichocarpa* were obtained from the Phytozome database (http://phytozome.jgi.doe.gov). Monolignol synthesis-related genes were identified in *S. matsudana*^[13]^, *S. suchowensis*^[14]^, and *S. purpurea* using genes from the six species above as queries in local BLAST searches (E-value < 1e^−10^) against the genomes of the three *Salix* species^[15]^. HMMER (http://www.hmmer.org) was used to determine whether the candidate genes contain the essential domains of each family^[16]^. The identified genes analyzed are listed in Supplemental Table S1.

### Phylogenetic analysis

Multiple protein sequences of the members in each gene family were aligned using ClustalW. A phylogenetic tree for each family was constructed using the Maximum-Likelihood method in MEGA X with the pairwise deletion option, the Poisson correction model^[17]^, and 1000 bootstrap replicates. Different clades within each family are identified using different colors.

### Chromosomal locations of lignin biosynthesis genes in *S. matsudana*

The chromosomal locations of all monolignol biosynthesis genes were obtained from the *S. matsudana* genome sequence database, and Mapchart 2.2 was used to generate a chromosomal location map^[18]^. Gene duplication events were defined based on two criteria: the alignment region covered more than 80% of the longer gene, and the identity of the aligned regions was greater than 80%. Regions that contained five or more lignin-related genes in less than 5 Mb were considered to be lignin biosynthesis hotspots^[17]^.

### Gene expression analysis and construction of a lignin biosynthesis gene network

We obtained cuttings from two *S. matsudana* parents with significantly different tree heights (TH) and diameters at breast height (tall and thick ‘9901’ and short and thin ‘Yanjiang’)^[19]^ and from the tall and thick F1 progeny ‘FH’ and the short and thin F1 progeny ‘FS.’ The cuttings (10 cm in length and 1 cm thick) were placed into nutrient soil in three replications. Stem terminals (0–5 cm long) were collected from each replication after 4 months and used for RNA-seq to measure the expression levels of monolignol synthesis genes in the four genotypes. Total RNA was extracted from three biological replicates of each genotype using the Plant RNA Reagent kit (Tiangen, China) according to the manufacturer's instructions. RNA was quantified using a NanoDrop ND 2000 spectrophotometer (Thermo Fisher, USA) and stored at −80 °C prior to sequencing on the Illumina platform (Illumina, USA) by Majorbio (Shanghai, China). RSEM (http://deweylab.github.io/RSEM) was used to calculate gene expression level as the fragments per kilobase of transcript per million mapped reads (FPKM), thereby normalizing for transcript length and the total number of mapped reads^[20]^. qRT-PCR analysis of the candidate genes was conducted using the One-Step SYBR Primer Script Plus RT-PCR kit (Takara, China) according to the manufacturer’s instructions. The *Actin* gene was used as an internal control^[15]^. All primers are listed in Supplemental Table S2.

Gene Ontology (GO) (http://www.geneontology.org) and KEGG pathway analyses were performed to assign potential functions to the assembled genes using Blast2GO^[21]^. The top three hits (*P* < 0.001) were used to provide an annotation for each target gene. Differentially expressed genes (DEGs) were defined based on two criteria using the DESeq2 package: (1) a two-fold difference in expression level (FPKM) between tall and short plants, and (2) a Benjamini–Hochberg FDR-adjusted *P-*value < 0.05^[22]^. The DEGs were visualized using heat maps constructed with the pheatmap2 function in R (version 4.0.1).

### Measurement of lignin content

Cuttings of the taller ‘9901’ parent, the shorter ‘Yanjiang’ parent, the tall F_1_ progeny ‘FH,’ and the short F_1_ progeny ‘FS’ (10 cm in length and 1 cm thick) were placed into nutrient soil in three replications. Stem terminals (3–8 cm from the top) were collected from each replication after 4 months. The samples were dried to a constant weight at 65 °C, then crushed, filtered through a 300–500 μm sieve, and weighed (W, dry weight). We then used the dried tissue to perform acetylation reactions using a lignin content test kit (Solarbio, Beijing, China) according to the manufacturer's instructions. Finally, OD280 (A) was measured using a UV spectrophotometer. The total lignin content was calculated using the following formula: lignin content (mg/g) = 2.184 × ΔA/W.

### Search for upstream sequence elements

For promoter analysis, 2000 bp of genomic DNA sequence upstream of the start codon (ATG) of each DEG was downloaded from the genome sequence database. Differentiating xylem–specific lignin biosynthesis genes were identified based on homology to *P. trichocarpa* differentiating xylem–specific genes in the AspWood database (http://aspwood.popgenie.org/aspwood-v3.0) using BLAST searches^[23]^. The PlantPAN 3.0 database (http://plantpan.itps.ncku.edu.tw)^[24]^ and ExactSearch, a fast plant motif search tool (http://sys.bio.mtu.edu/motif)^[25]^, were used to search for *cis*-acting regulatory elements in the putative promoter regions. A heatmap was drawn using the matrix2png interface (https://matrix2png.msl.ubc.ca)^[26]^.

## RESULTS

### Identification and comparison of monolignol biosynthesis genes from nine species

To compare monolignol biosynthesis genes in angiosperms, nine species (*A. thaliana*, *O. sativa*, *Z. mays*, *S. lycopersicum*, *S. suchowensis*, *S. purpurea*, *P. euphratica*, *P. trichocarpa*, and *S. matsudana*) were selected for phylogenetic analysis. These species could be divided into four groups: herbaceous monocots (*O. sativa* and *Z. mays*), herbaceous dicots (*A. thaliana* and *S. lycopersicum*), shrubs (*S. suchowensis* and *S. purpurea*), and arbors (*P. euphratica*, *P. trichocarpa*, and *S. matsudana*). The basic biosynthetic pathway of monolignol has been described previously^[27,28]^. Eleven gene families encoding enzymes of phenylpropanoid biosynthesis that participate in monolignol synthesis are as follows: *PAL*, *C4H*, *4CL*, *HCT*, *C3H*, *CSE*, *COMT*, *F5H*, *CCoAOMT*, *CCR*, and *CAD*. These 11 gene families were used for phylogenetic analysis. Sequence searches at the Pfam database were used to confirm the presence of specific protein domains in the predicted proteins of these genes (Supplemental Table S3).

The number of genes in each family differed markedly among the nine species (Table 1). The total number of monolignol biosynthesis genes in each species ranged from 44 to 192. *A. thaliana* had the lowest number, consistent with its small and simple genome. The total number of lignin-related genes in *S. matsudana* was nearly twice that of the *Populus* species or the shrub willows. This may be because *S. matsudana* is a tetraploid, and the other species are diploids. As expected, herbaceous plants contained fewer lignin biosynthesis genes than woody plants in the angiosperms. The total gene numbers in herbaceous monocots and herbaceous dicots were similar. It is not surprising that *S. lycopersicum* contained more lignin-related genes than *Z. mays*, *O. sativa*, and *A. thaliana*, as *S. lycopersicum* encompasses both herbaceous and woody varieties. However, the *Populus* species and the shrub willows contained similar numbers of genes, with *S. purpurea* containing more genes than the *Populus* species. These results indicate that woody plants contain substantially more lignin biosynthesis genes than herbaceous plants in the angiosperms, but there is little difference in lignin-related gene numbers between shrubs and arbors.

**Figure 1:**
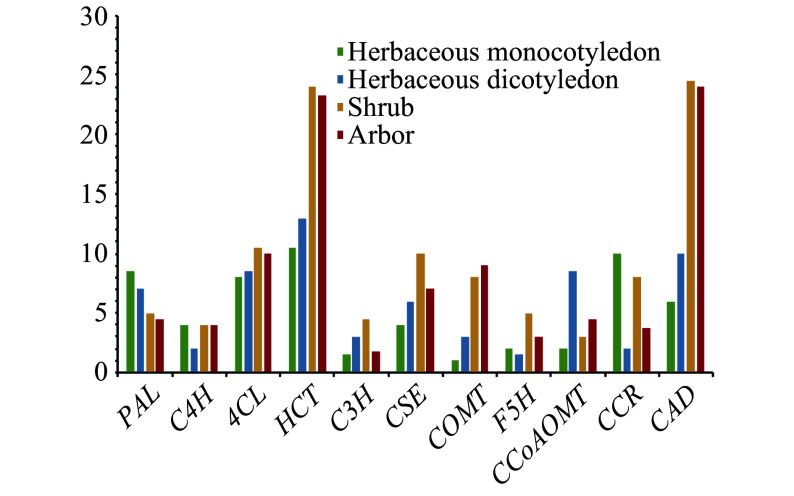
The average gene number in each lignin biosynthesis gene family in herbaceous monocots, herbaceous dicots, shrubs, and trees.

We compared the average gene number in each family among herbaceous monocots, herbaceous dicots, shrubs, and arbors. To minimize the effect of tetraploidy in *S. matsudana*, we divided its genes into the At and Bt sub-genomes based on their homology to *P. trichocarpa* genes, as previously reported^[13]^. As expected, several families showed marked differences in gene number among the four plant groups (Fig. 1). The *HCT*, *COMT*, *CAD*, *F5H*, *4CL*, and *CSE* families contained more members in woody plants than in herbaceous dicots. The *COMT*, *HCT*, *CAD*, *F5H*, *4CL*, and *CSE* gene families may therefore have undergone expansion or contraction during the evolution of herbs and woody plants in angiosperms, whereas other families showed no such trends.

### Phylogenetic analysis of 11 gene families

**Figure 2:**
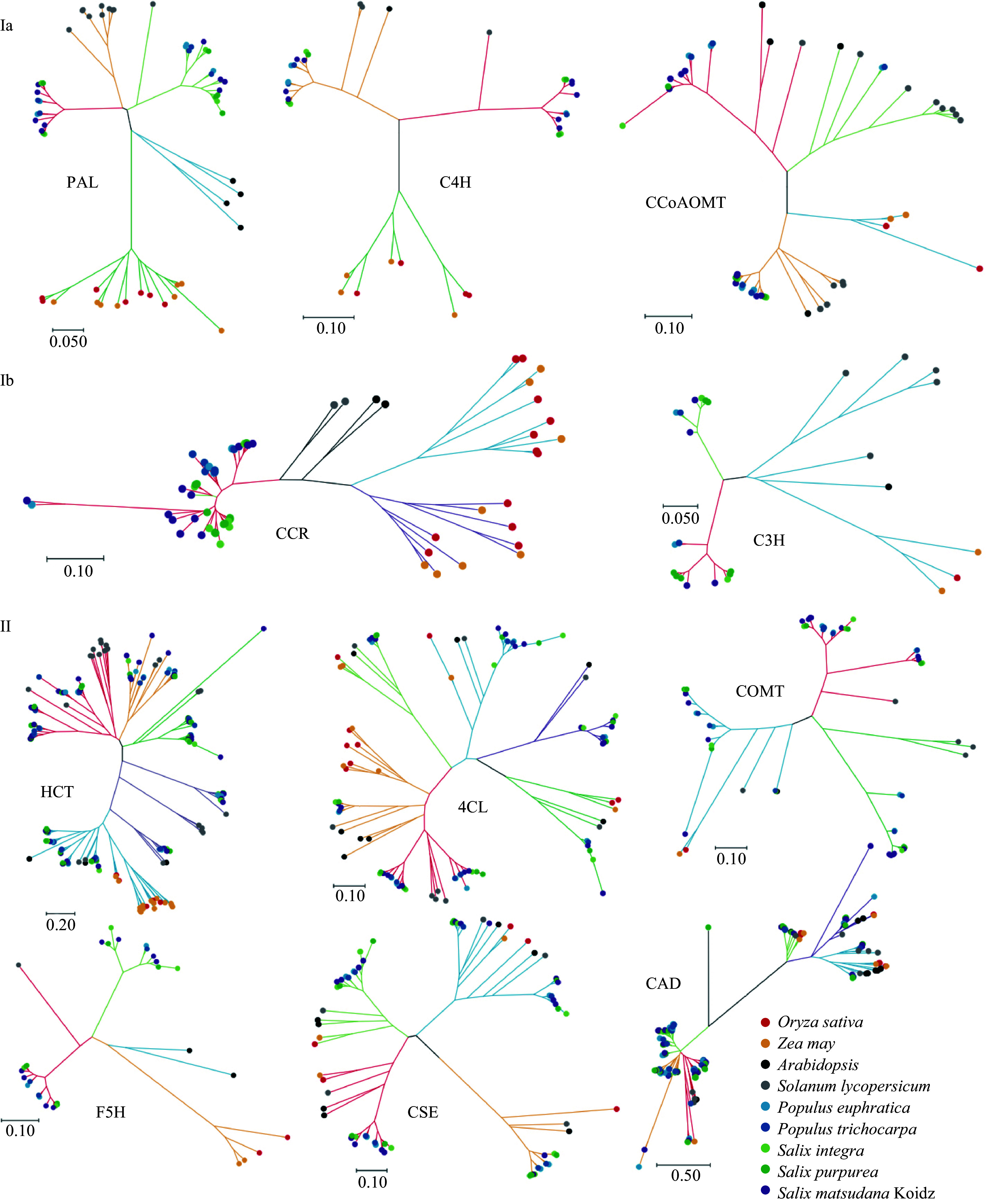
Phylogenetic trees of lignin biosynthesis genes from *A. thaliana*, *O. sativa*, *Z. mays, S. lycopersicum*, *S. suchowensis*, *S. purpurea*, *P. euphratica*, *P. trichocarpa*, and *S. matsudana*. The trees were constructed using MEGA X with the neighbor-joining method and 1000 bootstrap replicates. Different colored lines indicate different groups of gene families.

We performed phylogenetic analyses for each gene family to study the evolutionary relationships among lignin biosynthesis-related genes. Based on phylogenetic trees of sequences from herbaceous monocots, herbaceous dicots, shrubs, and arbors, the 11 gene families could be divided into two classes that differed in their apparent evolutionary histories (Fig. 2). Class I contained “differentiated gene families” and could be further divided into two sub-classes, Ia and Ib. Class Ia contained the *PAL*, *C4H*, and *CCoAOMT* families, and genes from monocots and dicots could be clearly distinguished. Genes in Class Ia appeared to have differentiated since the divergence of monocots and dicots. Class Ib contained the *CCR* and *C3H* gene families. Genes in this class were divided into three lineages belonging to the herbaceous monocots, the herbaceous dicots, and woody plants. These gene families appeared to have differentiated twice since the appearance of woody plants and monocots. Class II contained “expanded gene families,” as genes from the nine species could be detected in nearly all sub-groups of these gene families (Fig. 2). The *HCT*, *4CL*, *CSE*, *CAD*, *F5H*, and *COMT* families belonged to this class. This prompted us to propose the following hypothesis: most duplication-induced new genes regulate lignin composition rather than lignin yield. As plants differentiated into herbaceous and woody plants, some lignin-related gene families may have expanded to produce a much more complex metabolic network.

To further explore this hypothesis, we compared the functions of lignin biosynthesis genes in Classes I and II. Gene families in Classes Ia and Ib catalyze the conversion of phenylalanine to monolignol, whereas most gene families in Class II regulate the production of specific monolignol varieties such as hydroxy-coniferyl alcohol and sinapyl alcohol. These results indicate that the Class I gene families may contribute in ensuring the completion of the monolignol biosynthesis pathway. By contrast, the expanded gene families of Class II contribute to abundant lignin production. However, there are some exceptions: *CCoAOMT* in Class Ia and *4CL* and *CAD* in Class II. A possible explanation is that other pressures may affect the evolution of these gene families. Given the low number of Class II genes in herbaceous plants, woody plants may produce more G-type and S-type lignin, consistent with a previous report that H-type lignin is more prevalent in herbaceous than woody plants in angiosperms^[29]^.

Two sub-classes could be defined within Class I. They differed in the position of the herbaceous dicot sequences, which were grouped with those from woody plants in Class Ia but grouped separately in Class Ib. It was clear that Class Ib further differentiated as dicots evolved. However, none of the 11 gene families could distinguish shrubs from arbors. This may be due to the lignin biosynthesis pathways in shrubs and arbors being similar.

### Chromosomal distribution of monolignol biosynthesis genes in *S. matsudana*

**Figure 3:**
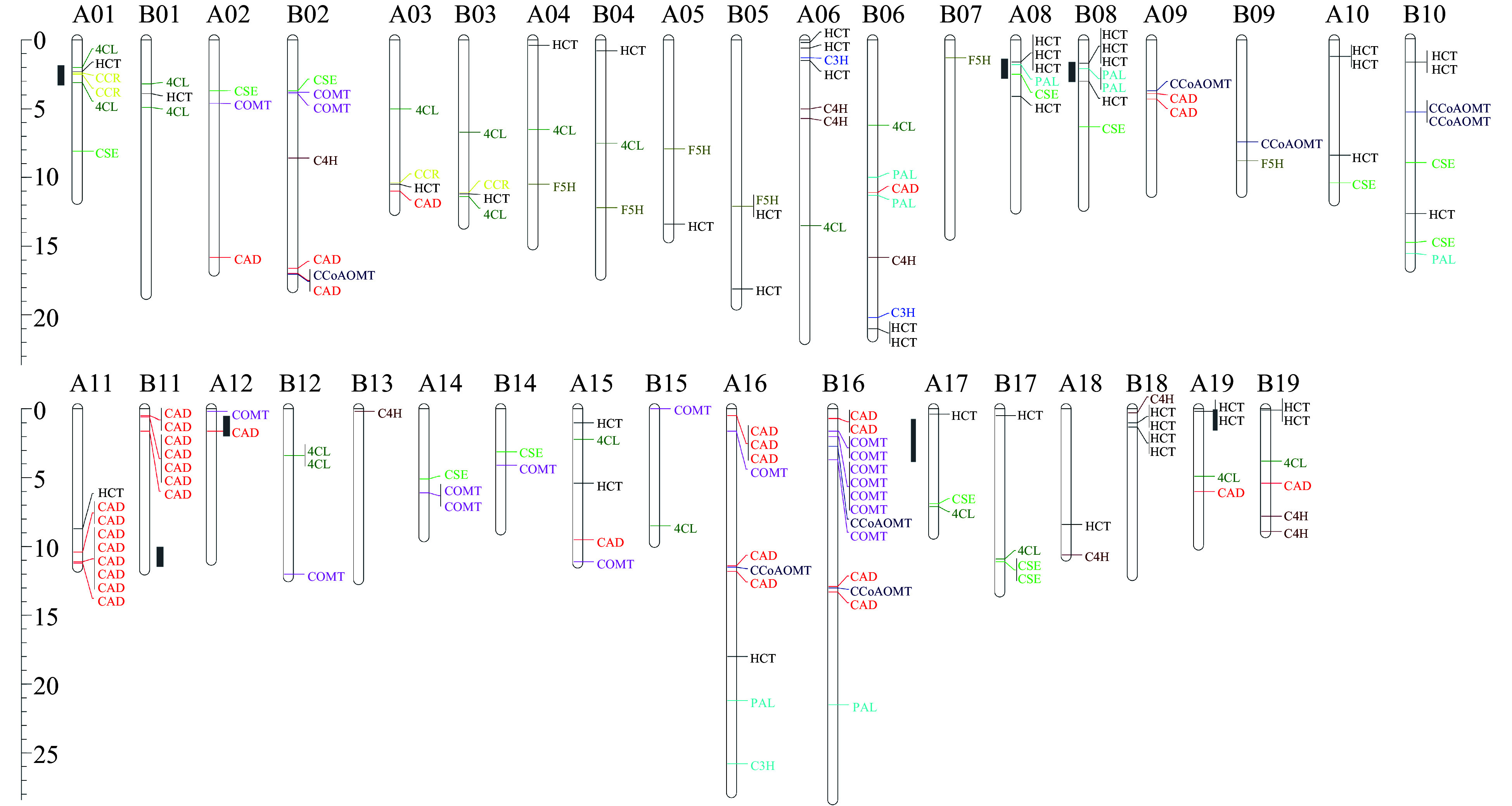
Distribution of lignin biosynthesis genes on *S. matsudana* chromosomes. The scale indicates megabases (Mb). Different gene families are indicated by different colors, and hotspots for lignin biosynthesis genes are marked in gray on the left of the chromosomes.

We mapped all identified lignin-related genes onto the genome of *S. matsudana*. 166 of the 192 genes could be mapped onto 36 chromosomes, and none were located on chromosomes A07 and A13 (Fig. 3). Chromosomes B16 and A16 contained the largest number of lignin-related genes. The At and Bt sub-genomes contained 76 and 90 lignin-related genes, respectively. However, 26 lignin-related genes could not be mapped onto the 38 *S. matsudana* chromosomes. Most genes in the *PAL*, *F5H*, *C4H*, *COMT*, and *CCoAOMT* families were located in the Bt sub-genome rather than the At sub-genome, whereas the At sub-genome contained more *CAD* and *CCR* genes. Among the 11 gene families, the *CAD* and *HCT* families had the largest number of members. Most *CAD* and *HCT* genes were present as tandem duplicates: *HCT* duplicates were found on chromosomes A08, B08, and B18, and *CAD* duplicates were found on chromosomes A11 and B11. We also identified a *COMT* cluster on chromosome B16, which may explain the abundance of *COMT* genes in the Bt sub-genome. Most homologous chromosomes exhibited good collinearity, although a few genes did not have corresponding alleles on homologous chromosomes. For example, *CCR* and *CSE* genes were detected on chromosome A01 but not B01, whereas *C4H* and *CCoAOMT* genes were identified on chromosome B02 but not A02. 17 and 31 genes were unique to the At and Bt sub-genomes, respectively. Based on the phylogenetic analysis of *S. suchowensis*, *S. purpurea*, and *S. matsudana*, 18 of these 48 genes occurred as tandem duplicates, such as *COMT* on chromosome B16 and *HCT* on chromosome B18. However, due to a lack of genomic information about their diploid ancestors, we could not determine whether these genes appeared before or after the appearance of *S. matsudana*.

### Lignin biosynthesis genes regulate the fast-growing trait of *S. matsudana*

To further understand the relationship between lignin biosynthesis and tree height (TH). We performed RNA-seq on stem terminals (0–5 cm long) from two parents (‘Yanjiang’ and ‘9901’) and two F1 progeny (‘FS’ and ‘FH’) to quantify the expression of lignin-related genes during primary stem growth. The expression levels of all 192 lignin-related genes were normalized to FPKM values. 117 genes had an FPKM > 1.0 in at least one biological replicate, and these genes were used for subsequent analysis. 23 of the 117 genes (19.66%) were differentially expressed between tall and short plants.

**Figure 4:**
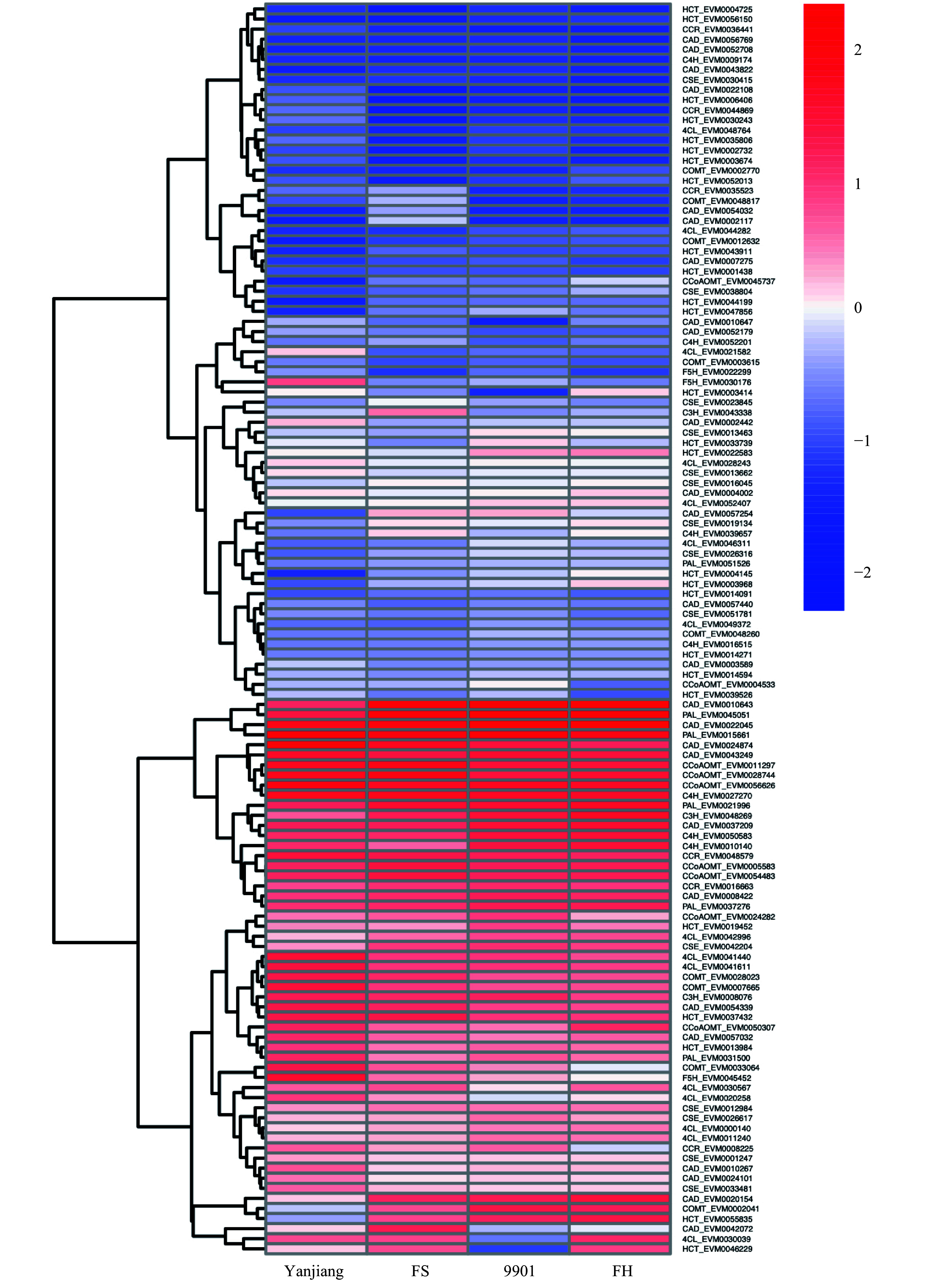
Expression profiles of 11 lignin biosynthesis gene family members in the two parents and two progenies. Red indicates a higher expression level, and blue indicates a lower expression level. Expression data are presented as FPKM values calculated in R.

**Figure 5:**
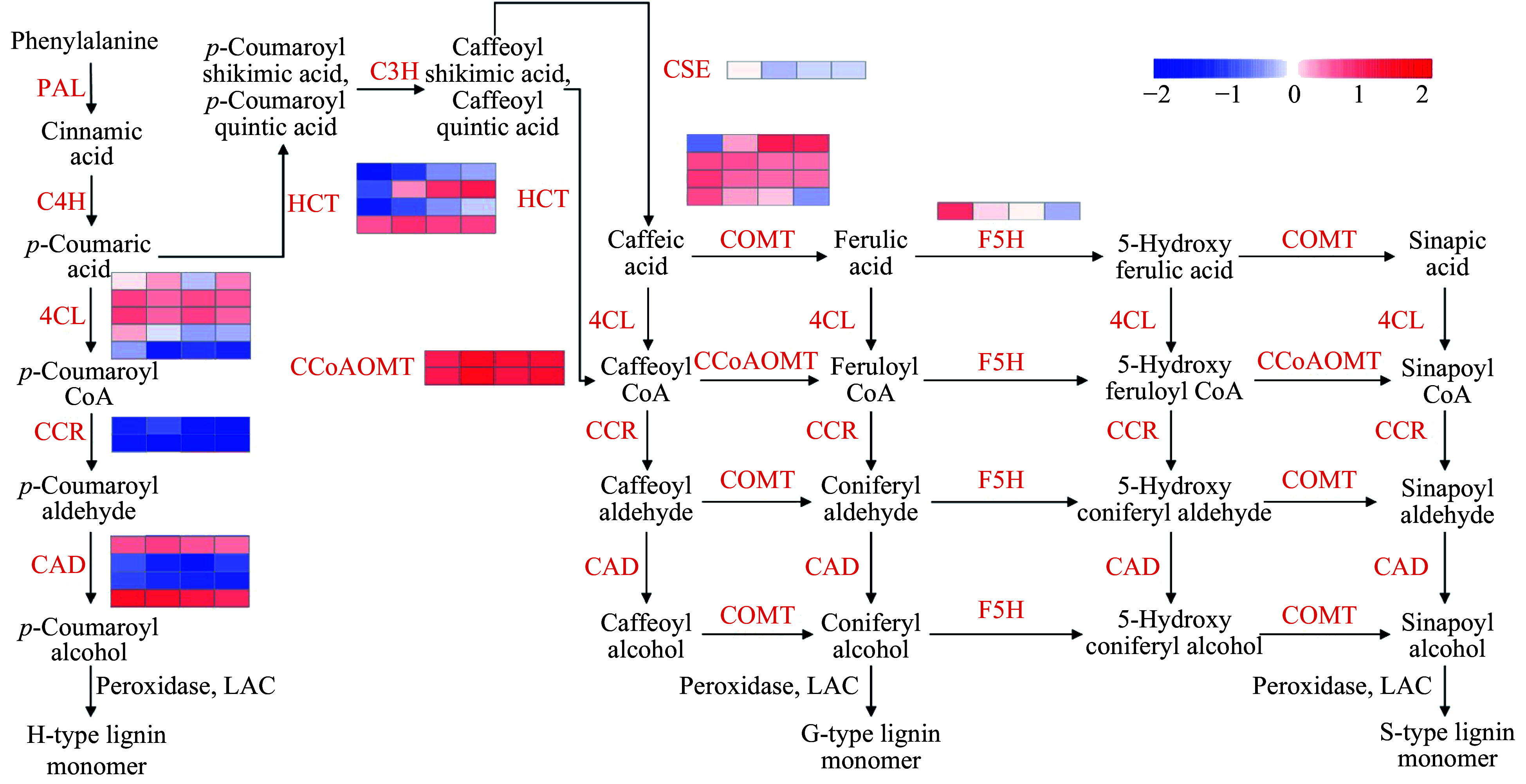
Gene expression network for monolignol biosynthesis in the stem terminals of *S. matsudana*. Transcript abundance (log_2_[FPKM+1]) in ‘Yanjiang’, ‘FS’, ‘9901’, and ‘FH’ is indicated by a color gradient.

Fig. 4 presents the expression profiles of 11 lignin-related gene families in *S. matsudana* stem terminals, based on the reference lignin biosynthesis model of *Arabidopsis* and *Populus* (Fig. 4). Overall, higher levels of lignin-related gene expression were observed in short plants compared with tall plants of *S. matsudana*. We identified 5, 4, 2, 2, 4, 1, 1, and 4 DEGs in the *4CL*, *CAD*, *CCoAOMT*, *CCR*, *COMT*, *CSE*, *F5H*, and *HCT* gene families, respectively. Four of the 23 DEGs were selected for qRT-PCR verification of differential gene expression, and the qRT-PCR results were consistent with the RNA-seq results (Supplemental Fig. S1). Four of the DEGs were expressed at a higher level in tall plants, and the other 19 DEGs were expressed at a higher level in short plants. With the exception of the *HCT* family, most gene families showed greater expression in short plants. We then measured the lignin content of the four genotypes. ‘9901’ and ‘FH’ contained less lignin than ‘Yanjiang’ and ‘FS’ (Table 2). The correlation between tree height and lignin content was *r* = −0.62 (*P* < 0.05). Based on the reference lignin biosynthesis model, we can infer that the expression levels of most lignin-related genes are negatively correlated with height (Fig. 5). By contrast, most *HCT* genes showed higher expression in the taller genotypes ‘9901’ and ‘FH’.

### Analysis of the promoter regions of lignin-related genes

**Figure 6:**
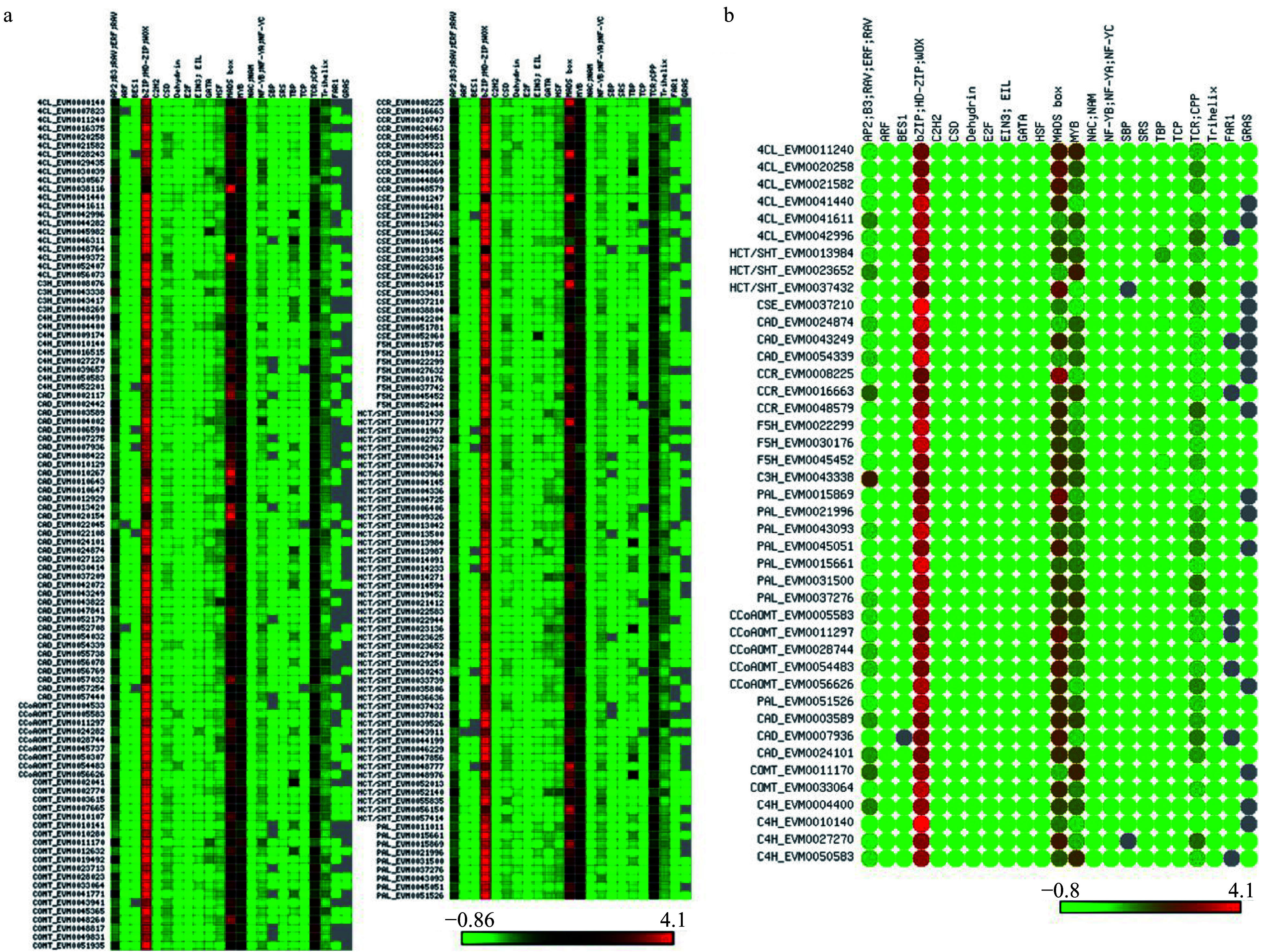
Motif analysis of promoter regions of the lignin biosynthesis genes. (a) Motif analysis of promoter regions from all *S. matsudana* lignin biosynthesis genes. (b) Motif analysis of promoter regions from differentiating xylem–specific lignin biosynthesis genes.

We identified *cis*-acting regulatory DNA elements within the promoter regions of all lignin-related genes using the PlantPAN database and ExactSearch. The gene promoters contained numerous DNA elements that are predicted to be bound by AP2, ARF, MADS-box, bZIP, NAC and MYB transcription factors (Fig. 6a). A total of 23 elements (motifs) were detected in the 11 families. As shown in Fig. 6a, the motifs showed little preferential distribution among different gene families. Genes from the same families often contained different motifs, and MYB, MADS-box, bZIP, AP2, and TCR-related motifs were detected on nearly all genes at a high frequency. The frequency of MYB- and AP2-related motifs showed substantial variation, and some motifs were not detected on a portion of the genes, such as FAR-and GRAS-related motifs. Interestingly, bZIP, MADS-box, and MYB related motifs were found in the upstream regions of nearly all 42 possible differentiating xylem–specific lignin genes, indicating that these motifs may participate in the regulation of lignin biosynthesis (Fig. 6b). This result is consistent with previous studies in which ethylene, cytokinin, and auxin-related transcription factors were reported to regulate lignin biosynthesis^[30−32]^.

## DISCUSSION

### The evolution of lignin biosynthesis-related genes in nine species

Fast growth is a complex quantitative trait that is controlled by environmental factors such as light, temperature, water, and fertilizer, although the effect of genetic variation is much greater. Among the traits related to fast growth in forest trees, height and diameter growth at breast height (DBH) are the most important. Tree height is determined primarily by the vessels, which are mainly composed of lignin, cellulose and hemicellulose in the secondary cell walls. Although the lignin biosynthesis pathway has been previously described^[27,28]^, differences in lignin biosynthesis between herbaceous and woody plants remains unclear. To further understand lignin biosynthesis in *S. matsudana*, we identified a total of 192 lignin biosynthesis genes in its genome and performed phylogenetic analyses using genes from nine angiosperm species, including herbaceous monocots, herbaceous dicots, shrubs, and arbors.

In this study, 11 gene families putatively related to monolignol biosynthesis were identified. Some of these 11 families may also function in flavonoid metabolism. For example, PAL, C4H, CCoAOMT, and 4CL also regulate the biosynthesis of flavonoids and terpenoid quinones^[33]^.

We could divide the genes of the monolignol biosynthesis pathway into two categories (Fig. 5). Genes in the first category catalyzed reactions that produced monolignol from phenylalanine, and most genes in Classes Ia and Ib could be placed in this category. Genes in the second category were involved in the production of specific monolignol varieties. Their encoded enzymes do not directly catalyze the production of monolignol from phenylalanine. Our phylogenetic analysis indicated that gene duplication had occurred primarily in Class II, which also contained more genes in woody plants than in herbaceous plants. These results are consistent with our hypothesis that expansions have occurred primarily in the families that participate in the regulation of lignin composition. However, *CCoAOMT* in Class Ia and *4CL* and *CAD* in Class II do not seem to conform to this hypothesis. This may reflect the fact that the evolution of these gene families is also influenced by other pressures.

Gene duplication is a major mechanism for the generation of new genes. After gene duplication, some duplicated genes undergo neofunctionalization, whereas others maintain largely redundant functions^[34]^. Duplicated genes exhibit various degrees of functional diversification in plants^[35]^. With the appearance of monocotyledons and dicotyledons, genes in Class I underwent differentiation. With the appearance of woody plants, Class Ib was further differentiated. For Class II, almost all gene families duplicated to generate new genes. However, more experiments or mutants are needed to infer the evolutionary fate of these diversified duplicates.

The remaining phylogenetic analysis showed that shrubs and arbors could not be clearly separated. The height and diameter of arbors are greater than those of shrubs, but their lignin biosynthesis genes are similar, although differences in the regulation of these genes may cause differences between the two growth forms.

### Distribution of lignin genes in *S. matsudana* At and Bt sub-genomes

Based on the sequenced genome of *S. matsudana*, 166 of the 192 identified lignin-related genes were located on 36 chromosomes. Gene numbers in the At and Bt sub-genomes were not equal. At and Bt contained 39.58% (76/192) and 46.88% (90/192) of all lignin genes, respectively. The At sub-genome was identified as homologous to the *P. trichocarpa* genome and contained fewer Class II genes than Bt. This result indicates that lignin components of *S. matsudana* and *P. trichocarpa* may differ. The At and Bt sub-genomes contained 8 and 11 DEGs, respectively. Several gene families were detected as duplicated on some chromosomes, such as *HCT* on chromosomes A08, B08, and B18, *CAD* on chromosomes A11 and B11, and *COMT* on chromosome B16. In summary, the Bt sub-genome contained more duplicated genes than the At sub-genome.

In this study, we also identified seven hotspots for lignin biosynthesis genes on chromosomes A01, A08, A11, B08, B11, B16, and B18 (Fig. 3). Most of the hotspots were located on the ends of chromosomes. Hotspots on chromosomes A01, A08, and A16 contained no less than three families. A comparison of the two sub-genomes would provide evidence on the evolution of *Salix* from diploid to tetraploid.

### Lignin biosynthesis genes and rapid growth

Tree height is mainly determined by primary growth. In a region extending down about 60 mm from the top of the tree, the stem begins to thicken gradually, and the secondary growth of vascular tissues begins^[36,37]^. We therefore sampled stem terminals (0–5 cm) from two parents and two F1 progeny of contrasting heights and performed RNA sequencing to detect the expression of lignin biosynthesis genes during primary growth. Nearly 60% (117/192) of the lignin-related genes were expressed at levels > 1.0 FPKM in the stem terminals, and 23 of these genes were differentially expressed between genotypes of different heights, suggesting that they may affect tree height by regulating lignin biosynthesis. qRT-PCR was performed to confirm the differential expression of the DEGs. Most of these genes were downregulated to suppress the biosynthesis of lignin in plants with tall genotypes (Fig. 5). However, in contrast to the other gene families, most differentially expressed *HCT* genes showed higher expression levels in tall plants. HCT catalyzes the conversion of coumaroyl-CoA to coumaroyl shikimic acid/coumaroyl quinic acid and the conversion of caffeoyl shikimic acid/caffeoyl quinic acid to coumaroyl-CoA. G subunits and S subunits are major components of lignin in dicots. Based on the reference lignin biosynthesis model, we can infer that the high expression of *COMT* promotes a high S/G ratio, which is currently used as a measurement standard in the paper industry. By comparing the transcriptomes of tall and short *S. matsudana*, we identified 23 DEGs involved in lignin biosynthesis. These genes may suppress plant height by promoting high lignin levels and regulating the S/G ratio. The high expression levels of the DEGs may also alter lignin content. In the paper industry, lower lignin contents and higher S/G ratios facilitate paper production^[38]^. However, further experiments are needed to determine whether the S/G ratio is associated with tree height.

Lignin is synthesized in the secondary cell walls of vessel cells and fiber cells and provides transporting function and mechanical support, respectively^[5]^. Previous studies have shown that the suppression of lignin biosynthesis in vessels can reduce plant growth, whereas the suppression of lignin biosynthesis in xylem fibers can improve biomass production without affecting plant growth^[39−42]^. Previous studies have reported that several transcription factors and hormones can regulate lignin biosynthesis^[30,43]^, and we therefore analyzed *cis*-elements in the 23 DEG promoters to better understand their regulation.

Previous studies found that the NAC-MYB module could regulate the expression of lignin biosynthesis genes^[44]^. In this study, we found that *cis* motifs were not distributed equally in genes from the same family, indicating that the regulation of these genes may also differ. Several hormone-related (auxin, ethylene, and cytokinin) transcription factor binding sites were identified in the promoter regions of the 23 DEGs. Previous studies have shown that trees have a radial gradient of auxin concentration within their secondary vascular tissues, with a maximum concentration in the cambium^[32]^. Unlike auxin, the distribution of cytokinins within secondary vascular tissues shows a peak in the developing phloem, although the two patterns partially overlap^[31]^. Tissue-specific transcriptome analysis in poplar showed that several genes related to auxin and cytokinin signaling and transport were differentially expressed during the regeneration of secondary vascular tissues, indicating that hormone distributions change during this process^[45]^.

A series of studies have shown that ethylene also participates in the growth and differentiation of cambial cells and affects the secondary growth of stems^[46−48]^. For example, in poplar, many ethylene biosynthesis enzymes and regulatory genes are expressed in woody tissues^[30]^. It has also been demonstrated that gravity can regulate ethylene content and cell division in cambium meristems, thereby affecting the formation of poplar tension wood^[49,50]^.

## CONCLUSIONS

We identified monolignol biosynthesis genes in the genome of *S. matsudana*. By comparing lignin-related gene families in nine plant species, we identified two patterns of gene family evolution in herbaceous monocots, herbaceous dicots, shrubs, and arbors. RNA-seq analysis from two parents and two F_1_ progeny enabled us to identify 23 DEGs that may participate in the regulation of accelerated growth. We also analyzed the distribution of lignin biosynthesis genes within the At and Bt genomes of *S. matsudana*. These results provide an important foundation for further studies on the molecular mechanisms and genetic regulation of lignin biosynthesis and the accelerated growth of forest trees.

**Table 1:** Numbers of genes in 11 lignin biosynthesis gene families from nine plant species.

Species	*PAL*	*C4H*	*4CL*	*HCT*	*C3H*	*CSE*	*COMT*	*F5H*	*CCoAOMT*	*CCR*	*CAD*
*Zea mays*	9	4	7	16	2	3	1	2	2	8	6
*Oryza sativa*	8	4	9	5	1	5	1	2	2	12	6
*Arabidopsis thaliana*	4	1	8	3	1	7	1	2	4	2	11
*Solanum lycopersicum*	10	3	9	23	5	5	5	1	13	2	9
*Salix suchowensis*	4	4	10	19	2	8	8	4	3	6	23
*Salix purpurea*	6	4	11	29	7	12	8	6	3	10	26
*Populus euphratica*	5	4	10	22	2	7	8	3	5	4	28
*Populus trichocarpa*	5	3	11	28	2	8	10	3	5	7	33
*Salix matsudana*	9	9	21	48	4	17	20	8	9	11	36
*Salix matsudana* At	2	3	8	22	2	6	6	2	2	3	20
*Salix matsudana* Bt	6	6	11	21	1	7	12	4	6	1	15

**Table 2:** Tree height and lignin content of ‘Yanjiang’, ‘FS’, ‘9901’, and ‘FH’.

	Height (cm)	Lignin content (mg/g)
Yanjiang	120.19 ± 7.67	1011.11 ± 205.52
FS	71.93 ± 5.13	633.33 ± 50.41
9901	153.69 ± 2.47	341.11 ± 15.48
FH	187.84 ± 3.24	255.56 ± 38.90

## SUPPLEMENTARY DATA

Supplementary data to this article can be found online.
