# Supplementary figures and images for: Genome-wide identification and analysis of monolignol biosynthesis genes in Salix matsudana Koidz and their relationship to accelerated growth
Source: For Res (Fayettev). 2021 Apr 23;1:8. doi: 10.48130/FR-2021-0008 (PMC11524315; doi:10.48130/FR-2021-0008)

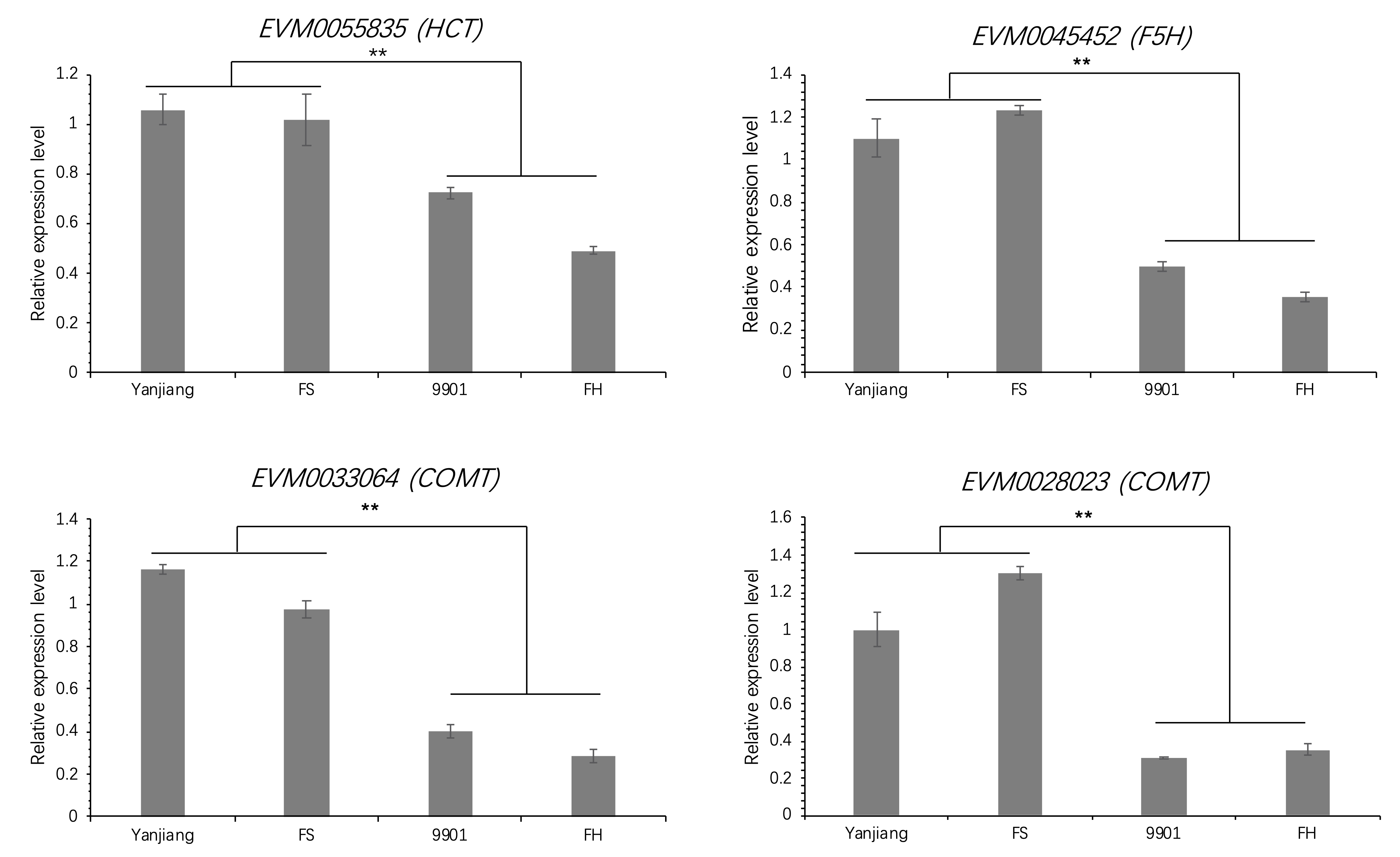

Supplement: Supplementary file 1 — Supplementary data to this article can be found online. [file FR-2021-0008-S1.zip › 10.48130_FR-2021-0008-Suppl-FigureSl.jpg]
